# Osteosphere Model to Evaluate Cell–Surface Interactions of Implantable Biomaterials

**DOI:** 10.3390/ma14195858

**Published:** 2021-10-07

**Authors:** Ana Carolina Batista Brochado, Victor Hugo de Souza, Joice Correa, Suzana Azevedo dos Anjos, Carlos Fernando de Almeida Barros Mourão, Angelo Cardarelli, Pietro Montemezzi, Vinicius Schott Gameiro, Mariana Rodrigues Pereira, Elena Mavropoulos, Gutemberg Gomes Alves

**Affiliations:** 1 Post-Graduation Program in Science & Biotechnology, Institute of Biology, Fluminense Federal University, Niteroi 24210-201, Brazil; anacarol.batista.b@gmail.com (A.C.B.B.); vh_souzalima@hotmail.com (V.H.d.S.); 2 Clinical Research Unit, Antônio Pedro University Hospital, Fluminense Federal University, Niteroi 24033-900, Brazil; joicecorrea@id.uff.br (J.C.); m.montemezzi@libero.it (P.M.); drschott@bol.com.br (V.S.G.); 3 Brazilian Center for Physics Research, Rio de Janeiro 22290-180, Brazil; elena@cbpf.br; 4 National Institute for Metrology, Standardization and Metrological Quality, Xerém 25250-020, Brazil; anjos.biologia@gmail.com; 5 Department of Dentistry, University Vita Salute San Raffaele, 20100 Milan, Italy; angelo_cardarelli@libero.it; 6 Neurobiology Department, Institute of Biology, Fluminense Federal University, Niteroi 24210-201, Brazil; rodriguespereira@gmail.com; 7 Cell and Molecular Biology Department, Institute of Biology, Fluminense Federal University, Niteroi 24210-201, Brazil

**Keywords:** tridimensional cell culture, osteoconductivity, biocompatibility, bone, biomaterials, titanium

## Abstract

Successful biomaterials for bone tissue therapy must present different biocompatible properties, such as the ability to stimulate the migration and proliferation of osteogenic cells on the implantable surface, to increase attachment and avoid the risks of implant movement after surgery. The present work investigates the applicability of a three-dimensional (3D) model of bone cells (osteospheres) in the evaluation of osteoconductive properties of different implant surfaces. Three different titanium surface treatments were tested: machined (MA), sandblasting and acid etching (BE), and Hydroxyapatite coating by plasma spray (PSHA). The surfaces were characterized by Scanning Electron Microscopy (SEM) and atomic force microscopy (AFM), confirming that they present very distinct roughness. After seeding the osteospheres, cell–surface interactions were studied in relation to cell proliferation, migration, and spreading. The results show that BE surfaces present higher densities of cells, leaving the aggregates towards than titanium surfaces, providing more evidence of migration. The PSHA surface presented the lowest performance in all analyses. The results indicate that the 3D model allows the focal analysis of an in vitro cell/surfaces interaction of cells and surfaces. Moreover, by demonstrating the agreement with the clinical data observed in the literature, they suggest a potential use as a predictive preclinical tool for investigating osteoconductive properties of novel biomaterials for bone therapy.

## 1. Introduction

Bone therapy in dentistry usually relies on the use of biomaterials from different origins, including xenografts, autografts, and allografts. Although autogenous grafts are, in many cases, the first treatment option, they have limitations as to their availability, and include the possibility of morbidity at the donor site. Therefore, the development of synthetic biomaterials that have a low cost and that can improve the quality of life of patients is of paramount importance [1]. The main synthetic bone graft materials available today consist of metals, polymers, ceramics, and composites [2]. Metallic biomaterials stand out for having good mechanical performance, being generally resistant to traction, fracture, fatigue, and corrosion [3]. Among metallic biomaterials, titanium (Ti) is more widely used in orthopedics and dentistry due to its greater resistance to corrosion and better biocompatibility [4,5].

In orthopedic applications, surface characteristics of titanium implants are critical in the clinical outcomes, by influencing biological responses, since micro and nanotopography impact the biological regulatory cascades involved in endosseous regeneration, and even on the microbiological control in implantology. Therefore, the success of a given material during bone therapy is directly related to its biocompatibility, and to properties such as osteoconduction, osteoinduction, and osteogenesis [6]. Osteogenesis and osteoinduction involve the ability of the material to induce a differentiation of mesenchymal cells, while osteoconduction is the ability to stimulate the attachment, migration, and growth of osteoblastic and osteoprogenitor cells, guiding bone growth along the material’s surface [7]. This is a fundamental process that leads to osteointegration, ensuring that the material remains fixed to the bone tissue; thus, avoiding the risks related to implant movement, which can generate inflammatory processes, necrosis, and rejection [8]. Therefore, it may be considered that osteointegration is determinant for the clinical success of an implant [9]. These biological properties depend on the physical and chemical properties of each material, such as the roughness, wettability, and surface load, which impact on cell–surface interactions [10]. The use of implants is not successful in many patients. In most cases, failure is associated with the low formation of bone tissue around the implant, which generates inflammatory processes that can lead to the need for a removal or replacement of the implanted material [11]. Therefore, modifications on the surface of Ti implants are usually performed to try to reduce the cases of failure [12]. One of the techniques used is treatment with sandblasting, followed by acid etching, which changes the nanotopography of the surface, generating macro roughness and micro porosity, resulting in an increase in the adhesion of osteoblasts [13]. Another technique used is coating with ceramic materials, such as hydroxyapatite (HA, Ca_5_(PO_4_)_3_(OH)). HA presents bioactivity in biological media and stimulates the production of hard tissue, increasing osseointegration [14].

The preclinical evaluation of osteoconductivity in biomaterial research currently involves animal experimentation with bone defect models, preceded by in vitro tests with a two-dimensional (2D) monolaminar cell culture. Nevertheless, in the context of in vitro assessments, new paradigms have arisen on the research of the biological effects of drugs and materials in contact with human tissues, with a major role for both in silico and in vitro assays, which are experiencing technological and scientifical breakthroughs to provide reliable and transferrable results to the clinics [15]. Such paradigms include the use of an improved cell system such as organoids and tridimensional models. The 2D model has many limitations and requires the constant research and development of new strategies, closer to the clinical and physiological models for testing new products. In this model, cells are devoid of a extracellular matrix (ECM), cell–cell interactions, and mechanical stimuli, which interfere with cell morphology and phenotype, and, consequently, limit the evaluation of the biological properties of some material [16]. In addition, this model has induced an apical–basal polarity that is not found in most cell types of in vivo tissue, which can change functions such as cell migration and spreading, fundamental phenomena for the analysis of the cell–material surface interaction [17]. The three-dimensional, organoid, and tissue-equivalent models have been considered extremely promising to increase the prediction of in vitro tests. Three-dimensional (3D) culture models, on the other hand, allow cells to interact with each other and with the ECM, interactions that control the physiological responses that occur in a tissue [18]. This allows cells to perform complex interactions with adjacent cells, receiving and transmitting signals. Furthermore, there is a reduction in stress and artificial results such as cell adaptation to flat surfaces such as in the 2D model [19].

While two-dimensional cell models have been widely used for biomaterial surface testing in numerous studies, there is little evidence of studies assessing the three-dimensional interactions that would be expected between a tissue and treated surfaces of implants, that could impact the quality of cell adhesion. In this context, the present study aims to characterize and evaluate the use of a three-dimensional bone cell model (osteospheres) in a methodology for the in vitro evaluation of cell–surface interaction of different titanium implant surfaces and investigate possible parameters that can contribute to the predictability of in vitro cell/surface analyses.

## 2. Materials and Methods

### 2.1. Titanium Surfaces and Surface Analysis

This work compared three different previously studied [20] surfaces for plateau root form (PRF) endosseous Ti-6Al-4V implants, provided by Bicon™ Dental Implants (Boston, MA, USA). The first corresponds to an “as machined” titanium surface (MA); the second is a blasted/acid-etched (BE) Ti-6Al-4V surface, blasted with alumina and passivated in a nitric acid solution (Integra-Ti; Bicon, Boston, MA, USA); the third is a Ti-6Al-4V surface with a 300–500 nm plasma sprayed hydroxyapatite coating (Integra-CP, Bicon, Boston, MA, USA), hereby identified as PSHA, for which an identification card and codification of the chemical and morphological characteristics are provided by Dohan Ehrenfest et al. [21]. The specimens consisted of cylinders 0.3 cm thick with 1.3 cm in diameter, and a treated surface area of 1.33 cm^2^. As a positive control of cell adhesion, Thermanox™ glass coverslips with 1.3 cm diameter were used (Thermo Fisher Scientific, Waltham, MA, USA).

### 2.2. X-ray Diffraction (XRD)

The titanium disc surfaces were characterized by XRD on an X-Pert Pro diffractometer (Panalytical, Westborough, MA, USA). International Center for Diffraction Data (ICDD) cards for titanium and hydroxyapatite were used to index the diffracted peaks of targets and films. The spectral peaks were extracted from the entire surface, corresponding to 1.33 cm^2^.

### 2.3. Fourier Transform Infrared Spectroscopy (FTIR)

Fourier transform infrared spectroscopy (FTIR) coupled to a microscope (AIM8800, Shimadzu, Kyoto, Japan) was used to assess the chemical composition of the surfaces. A Shimadzu IR-Prestige 21 (Shimadzu, Kyoto, Japan) equipment operating in attenuated total reflectance (ATR) mode was used. The measurement conditions were: IR beam incidence area of 432 µm × 332 µm, analyzed wavelength range of 500–4000 cm^−1^, beam resolution of 4 cm^−1^ and scan 300 times per sample.

### 2.4. Atomic Force Microscopy (AFM)

A NanoScope V system (Bruker, Karlsruhe, Germany) was used in contact mode at 1 Hz to assess parameters of surface roughness of each surface. The tips used (NT-MDT) had a 10 nm radius of curvature. The following parameters were analyzed with the Gwyddion v2.59 software: mean roughness (Sa), mean quadratic roughness (Sq), maximum pit depth (Sv), and maximum height (Sz).

### 2.5. Scanning Electron Microscopy (SEM)

The surfaces were observed through SEM. The discs were covered with a gold layer to avoid decomposition of the analyzed material. The analysis was performed using a Field Emission Gun-Scanning Electron Microscope (FEG-SEM) (JSM-7100F, JEOL, Tokyo, Japan).

### 2.6. Cell Culture and Production of Osteospheres

Human osteoblast-like MG-63 cells (Rio de Janeiro Cell Bank—BCRJ) were maintained in Dulbecco’s Modified Eagle Medium–High Glucose (DMEM) supplemented with 10% fetal bovine serum (FBS) and 1% penicillin/streptomycin at 37 °C in a 5% CO_2_ incubator. The tests were performed using cells with 99% of cell viability in passages between 24 and 27. The liquid overlay technique was used to produce the aggregates. The 96-well conical bottom microplates were coated with 2% sterile agar (bacterial agar, Sigma-Aldrich, Sao Paulo, Brazil). A cell density of 2 × 10^4^ cells was seeded with 130 µL of high glucose DMEM medium supplemented with 10% FBS. The plates were incubated under agitation using an orbital shaker (MS-100, Thermo, Waltham, MA, USA) for 200 rpm at 37 °C and 5% CO_2_ for 4 days.

### 2.7. Osteospheres Characterization

The average diameter of the spheres was measured through the evaluation of micrographs taken by optical microscopy (ZEISS Axio A1, ZEISS, Jena, Germany), using the software Image-Pro Plus 6.0 (Media Cybernetics, Rockville, MD, USA). To estimate the average cell density, the spheroids were transferred to 0.5 mL microtubes (1 spheroid per microtube), washed twice with phosphate saline buffer (PBS) and, subsequently, 100 µL of disintegrating solution (2.5 mg/mL Trypsin from porcine pancreas (Sigma-Aldrich, Burlington, MA, USA)) and 1 mg/mL Collagenase from Clostridium histolyticum-type 1A (Sigma-Aldrich, Burlington, MA, USA) were added. The cells were stained with Trypan Blue and counted on a hemocytometer.

For the histological analysis, after 4 days of formation, the samples were frozen in tissue freezing medium (OCT-Killik, Easy Path, Somerville, MA, USA), subsequently, cut into 7 µm sections using cryostat (CM1850, Leica, Jena, Germany) and then stained with Hematoxylin and Eosin (H&E). To observe calcium deposition in the aggregate matrix, the sections were stained with 2% alizarin red. The images were obtained using a ZEISS Axio A1 optical microscope (ZEISS, Jena, Germany).

### 2.8. Exposure to the Titanium Surfaces

The titanium discs and the control surface (Thermanox, Thermo, Waltham, MA, USA) were placed in 24-well microplates (one per well). Cloning tubes with a diameter of 0.8 cm and an area of 0.5 cm^2^ were added on top of each disc to prevent the spheroids from rolling out of the materials. Spheroids were transferred from the culture plate to the disks using an automatic pipette with a volume of 20 µL. Each disc received 4 spheroids, placed on different quadrants of each disc surface, and 700 µL of high glucose DMEM supplemented with 10% FBS and 1% streptomycin/penicillin was added. The plates were incubated at 37 °C and 5% CO_2_ for a total of 7 days, and samples were collected on days 2, 5, and 7.

### 2.9. Qualitative Analysis of Aggregate-Material Interaction

The interaction of the spheres and the material surfaces was also assessed using scanning electron microscopy (SEM). Samples were fixed in a Karnovsky fixative (Paraformaldehyde 2.0 gm, 1M Sodium hydroxide, 50% glutaraldehyde and 0.2M cacodylate buffer, pH7.4) for 30 min, followed by three 5 min washes in sodium cacodylate buffer. Subsequently, post-fixation was performed, in which the samples were dehydrated in a series of ethanol solutions (15–100%), treated with 1:1 of ethanol and hexamethyldisilane (HMDS) for 10 min, followed by pure HMDS for 10 min. After the samples were completely dried, they were coated with a 20 nm thick gold layer and examined on a FEG-SEM electron microscope (JSM-7100F, JEOL, Tokyo, Japan) at 5 kV.

### 2.10. Total Cell Density Estimation

The density of cells present in each material was estimated by detecting the total protein content and the enzyme Lactate Dehydrogenase (LDH) released after total dissolution of cell membranes. After exposing the osteospheres to each surface for 2, 5, and 7 days, the materials were washed once with PBS to remove the non-adherent cells and then 400 µL of culture medium with 1% Triton X-100 was added to each well. The plates were incubated for 24 h and, after that period, 60 μL of the medium from each well was transferred to another plate, and 240 μL of the mixture of the reagents LDH II and LDH III (In CytoTox, Xenometrix, Sinsheim, Germany) was added. The Optical density (O.D.) was read in a microplate reader (Sinergy II, BioTek Inst., Winooski, VT, USA) for 60 min at 37 °C at 340 nm. The protein content was estimated in the same samples through a Bradford Protein Detection kit (Bio-Rad, Hercules, CA, USA), considering culture media with FBS as a discounted background.

### 2.11. Estimation of Cell Density on the Surface of the Material

The samples were fixed in PFA 4%, washed with PBS, and then exposed for 10 min to an ammonium chloride solution (20 mM). The cells were washed with PBS and then had their membrane permeabilized through Triton X-100 for 10 min. After washing again with PBS, they were immersed in 3% BSA for 10 min. The cells were marked with phalloidin conjugated to tetramethylrhodamine isothiocyanate or TRITC (1:100) for 30 min, to mark the actin cytoskeleton, and washed with PBS afterwards. Finally, the nuclear marker 4’, 6’-diamino-2-phenyl-indole, or DAPI, was added (1:5000) for 30 min and, after that time, the cells were washed again with PBS and stored in 4 mg/ml DABCO 1, 4-diazabicyclo [2.2.2] octane. The micrographs were taken from 10 different, not superposed, fields per disk with a 2.5 Mp camera (Axiocam, ZEISS, Jena, Germany) attached to the Fluorescence Microscope (ZEISS Axio A1, Zeiss, Jena, Germany). The quantification of the cells that adhered to the material was performed by quantifying the DAPI-stained nuclei of cells outside the cell aggregates present in the images using the Image-Pro Plus 6.0 software.

### 2.12. Estimation of Cell Migration

The samples were prepared, and the images obtained and analyzed as described in Section 2.10. The cells then left the aggregate to form a halo around it, allowing the analysis of cell migration and proliferation to be conducted by measuring the distance between the edge of the aggregate and the most distant cells leaving the spheroid. For each spheroid, the value considered the mean of 4 radium positions measured (left, right, top, and bottom).

### 2.13. Analysis of Cell Spreading

The samples were prepared, and the images obtained as described in Section 2.10. The images used were obtained at 40× magnification and the analysis was performed using the Image-Pro Plus 6.0 software. For analysis of cell spread, the total area covered by the submembrane actin network was measured and considered as representative of the cell size.

### 2.14. Statistical Analysis

The analyses were performed in biological and technical triplicates. The statistical tests were performed using the GraphPad Prism 8.0 software. Normal distribution was assessed thorough D’Agostino–Pearson omnibus tests, and non-parametric (Kruskal–Wallis) analysis of variance tests were performed with Dunn’s post hoc test, comparing all groups. The effects of time on cell density after seeding on the titanium surfaces were assessed by two-way ANOVA with a Tukey post hoc test. Results were considered significant with *p* < 0.05.

## 3. Results

### 3.1. Surface Characterization

#### 3.1.1. X-ray Diffraction (XRD)

**Figure 1:**
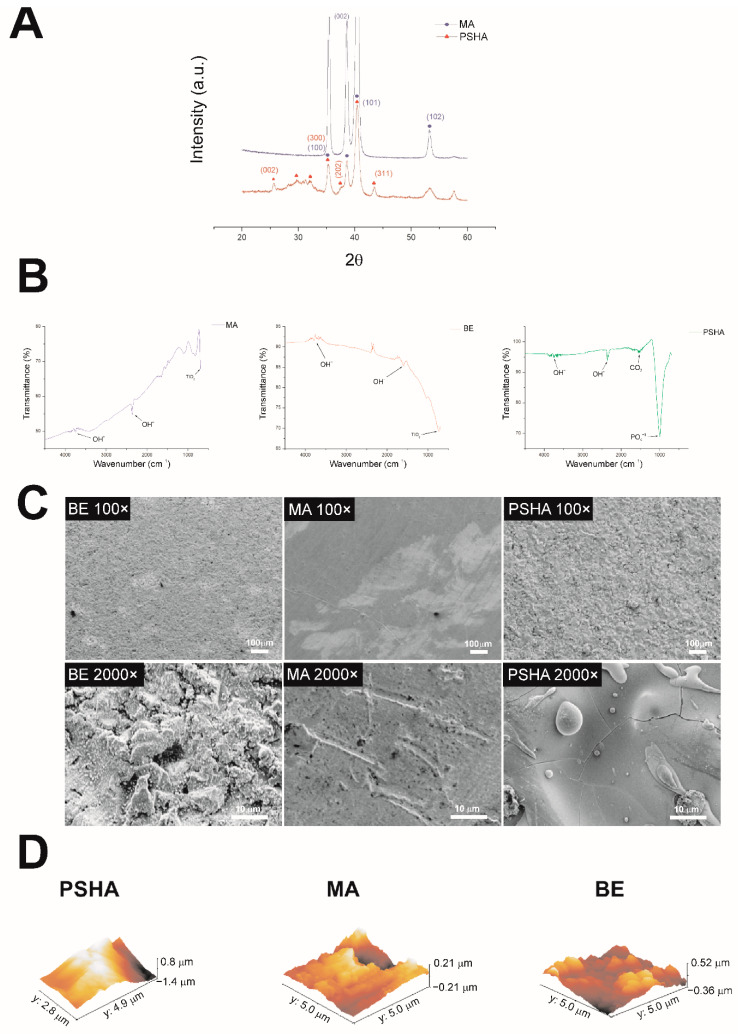
Surface analysis of the tested titanium samples. (**A**) XRD diffractogram of MA (blue) and PSHA (red) sample surfaces. (**B**) FTIR of machined surfaces (MA), sandblasting and etching (BE) and HA plasma spray (PSHA). (**C**) Electron micrographs of titanium surfaces with the three different types of treatment at two magnifications. (**D**) Atomic Force Microscope 3D views of the titanium surfaces.

The crystalline phases and order of hydroxyapatite of PSHA surface were evaluated by XRD. The machined surface (MA) showed similarities with the crystallographic profile present in the JCPDS-ICDD 44–1294 for titanium, which had the highest intensity peak at 40° related to the (101) plane; also, the lowest intensity peaks presented at 35° and 38° associated with planes (100) and (002) (Figure 1A). The titanium peaks in the diffractogram of the PSHA sample presented a lower intensity than the pure titanium samples, indicating the presence of a thin film. In addition, characteristic peaks of calcium phosphates, in the region between the angles 27° and 32°, appeared poorly defined, suggesting an amorphous characteristic of the coating. The diffractogram also showed a low-intensity, but well-defined peak close to 25.5°, that could suggest the presence of plane (002) in the JCPDS-ICDD 9-432.

#### 3.1.2. Fourier Transform Infrared Spectroscopy (FTIR)

Figure 1B shows that MA and BE surfaces presenting the characteristic peak of titanium at 690.52 cm^−1^ [9]. On the PSHA surface, the characteristic peak of PO4^−3^ was observed between 1000 and 1100 [10], and of CO_3_ in 1532 cm^−1^ [11].

#### 3.1.3. Scanning Electron Microscopy

The scanning electron microscopy of the surfaces showed topographic differences (Figure 1C). As expected, the MA surface showed less irregularity than the other two. The BE surface showed evident irregularities caused by sand jets and acid treatments. The PSHA surface had ceramic characteristics and a surface irregularity characteristic of the deposition of HA and calcium phosphates.

#### 3.1.4. Atomic Force Microscopy (AFM)

The analysis of different surface parameters by AFM (Table 1, Figure 1D) showed that the MA surface had a significantly lower roughness compared to the other two treatments (*p* < 0.05). The BE surface showed very high peaks and very deep valleys, with an intermediate roughness compared to the MA and PSHA groups, but with no significant difference between them (Table 1 and Figure 1D).

### 3.2. Spheroid Characterization

To select the best cell density for the formation of stable and consistent osteospheres, we analyzed the aspect (diameter and uniformity) of aggregates formed from three different initial cell densities (Table 2). All aggregates presented similar and adequate uniformity indexes with a height/width ratio of around 1.2, i.e., with a spheroid shape. The cell density of 20,000 cells showed a similar diameter to most three-dimensional models of the literature [22,23,24], and a final cell density, similar to the initially expected, of 1.9 × 10^4^ ± 0.32 four days after seeding.

**Figure 2:**
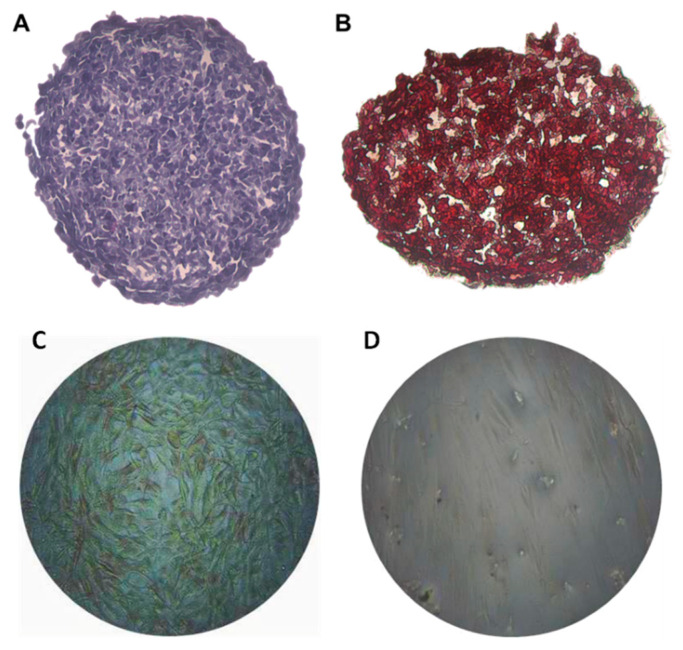
Histological sections of aggregates of 20,000 cells with 4 days of formation. (**A**) Sections stained with H&E. (**B**) Sections stained with alizarin red. (**C**) MG63 osteoblasts cultured in monolaminar 2D cell culture at day four, stained with alizarin red in the same culture conditions of the osteospheres, where the absence of significant staining can be observed. (**D**) Human gingival fibroblasts in monolaminar 2D cell culture, presented as a negative control for mineralization after staining with alizarin red.

The histological sections with H&E staining presented in Figure 2A showed very compact aggregates with a uniform cell distribution in all layers of the spheroids, spaces between the cells ranging from the surface to the central layers, and no evidence of a necrotic nucleus. The alizarin red S staining (Figure 2B) indicated that these aggregates already had calcium deposits on the fourth day of formation, suggesting evidence of mineralization of the matrix before being exposed to tests.

**Figure 3:**
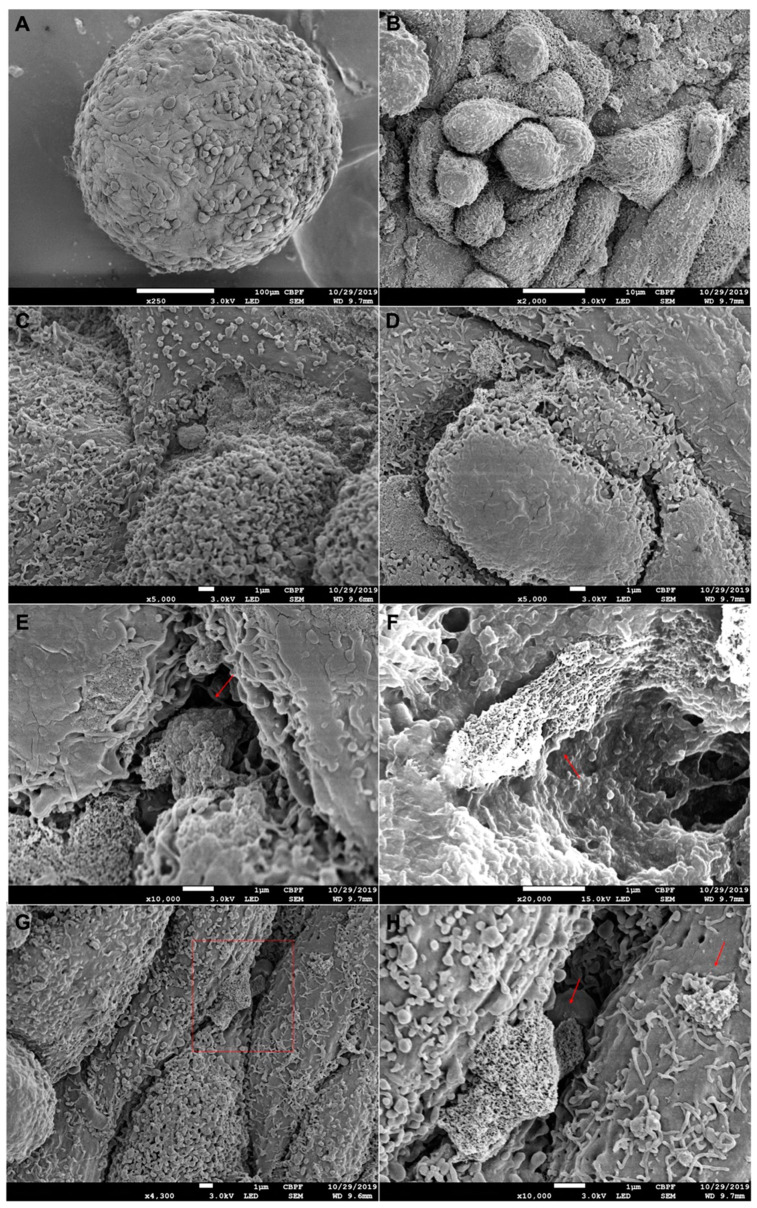
Electron micrographs of MG-63 osteospheres, 4 days of formation. (**A**) At 250× magnification. Higher magnification photomicrographs show cells with many microvilli and strong intercellular interaction on the aggregate surface (**B**–**D**). It is also possible to observe spaces between the cells and the formation of deposits on the cell surfaces that enter the aggregate, which is indicated by the red arrows in the figures (**E**–**G**). Figure (**H**) shows a larger magnification of the red square region in Figure 3G.

The scanning electron microscopy (SEM) analysis showed a uniform morphology (Figure 3A) with remarkable intercellular interactions, cells with many microvilli and morphological heterogeneity on the surface (Figure 3B–D). It was possible to observe spaces between cells, and the formation of deposits extending to the innermost layers in all analyzed spheroids (Figure 3E,F,H).

### 3.3. In Vitro Analysis of Cell/Surface Interactions

#### 3.3.1. Qualitative Evaluation of Cell Adhesion

**Figure 4:**
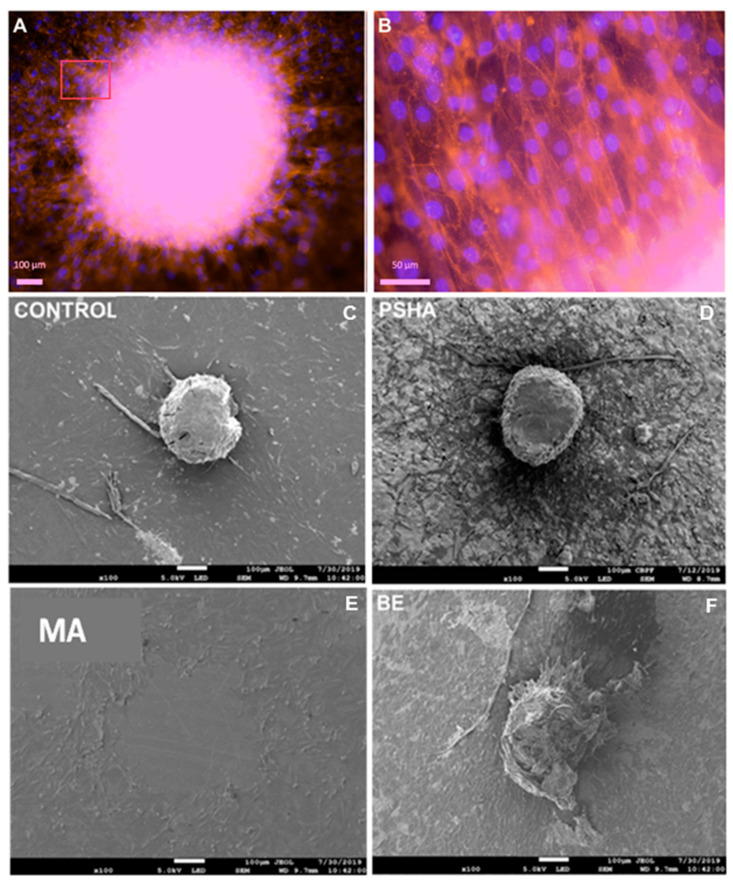
Images of the interaction of the osteospheres with the different material surfaces by fluorescence microscopy (**A**,**B**) and scanning electron microscopy (**C**–**F**). Cells migrating and adhering to the control surface (Thermanox) at 20× magnification (**A**). The region indicated by the red quadrant is shown at higher magnification in (**B**). The cells have the cytoskeleton stained with phalloidin in red and the nucleus marked with DAPI in blue. Panels C to F show SEM micrographs of aggregates adhered to the control and titanium surfaces with 5 days of exposure. A pattern of cells can be seen leaving the aggregates and covering the material surfaces, even when the aggregate was unattached during sample processing (MA).

Figure 4 shows osteospheres adhered to the different titanium surfaces. In the MA surface, most aggregates unattached from the materials after sample processing, but it was still possible to observe their original place of adherence through the halo of remaining cells around the initial adhesion area. In all surfaces, it was possible to observe that cells left the spheroid towards the titanium surface, forming a “solar” pattern around the aggregate. The BE surface appeared to have a greater cell density around the osteospheres, as did the control. The BE surface aggregate showed signs of dismantling, with an irregular and disorganized morphology.

#### 3.3.2. Quantitative Evaluation of Cell Density

**Figure 5:**
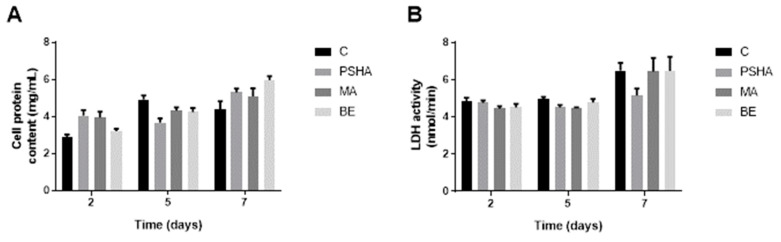
Estimation of total cell density by total protein content (**A**) and LDH enzyme activity (**B**) and total protein after membrane solubilization. No significant difference was found between groups (*p* > 0.05), but growth over time was significantly different at 7 days (*p* < 0.05). Bars indicate mean ± SEM of three independent tests with five biological replicates each.

Through estimating the cell density on each surface by the total protein and enzyme activity (Figure 5), it was possible to observe that, over time, the cell density increased and became significantly different at 7 days (*p* < 0.05). However, there was no significant difference between the groups studied on any of the days evaluated. It is important to note that using this method, the cells counted included the cells adhered to the surface plus the cells that constituted the aggregates.

**Figure 6:**
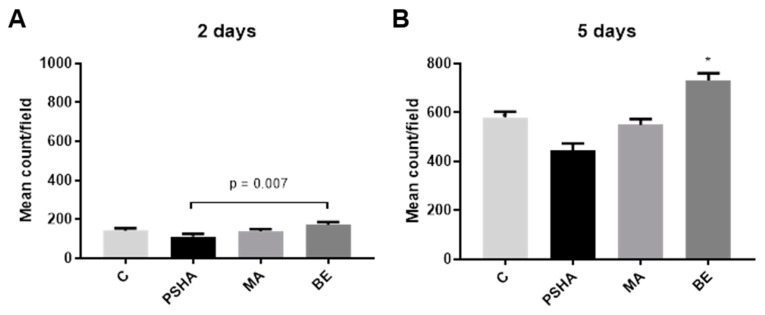
The counting of cells adhered to the surface through fluorescence showed that in 2 days (**A**) there was a significant difference only between the BE and PSHA groups with *p* = 0.007. On day 5 (**B**), the BE group had a significantly higher number of cells compared to the other groups (BE vs. C *p* = 0.0006; BE vs. PSHA *p* < 0.0001; BE vs. MA *p* < 0.0001), and the PSHA group had significantly lower cell counts than all the other groups (PSHA vs. C *p* = 0.003; PSHA vs. MA (*p* = 0.028; PSHA vs. BE *p* < 0.0001). Bars indicate mean ± SEM of three independent tests with five biological replicates each. * Indicates significant difference from all other groups (*p* < 0.05).

A quantitative analysis using the fluorescence method was not possible on the seventh day, as the cells had practically covered the entire surface of the materials, presenting overlapping cells, which made it impossible to delimit the cytoplasm and nuclei. As can be seen in Figure 6, with 2 days of exposure to the surfaces, the BE group had significantly higher amounts of adhered cells outside the aggregate than the PSHA group (*p* = 0.007). On the fifth day, we observed an increase in cells adhering to surfaces in all groups. When comparing the different surfaces, the number of cells was significantly higher in the BE surface, and significantly lower in the PSHA group (*p* < 0.05).

#### 3.3.3. Cell Dispersion on Titanium Surfaces

**Figure 7:**
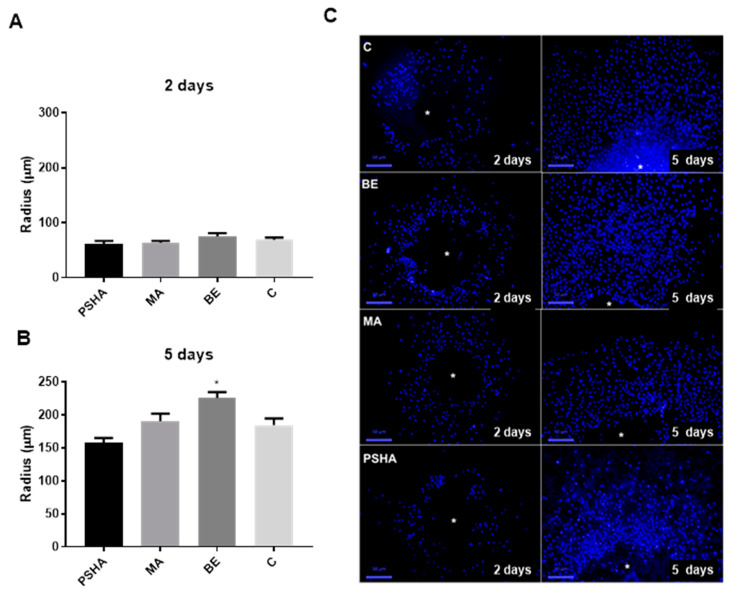
Estimation of migration/proliferation over the titanium surfaces, through the measurement of the radius of the distance between the edge of the aggregate and the most distant cells in 2 days (**A**) and 5 days (**B**). (**C**) The cell halo which was seen when aggregate was removed due to sample processing and which was used for quantitative analysis. This is evident that cell density increased with the time of incubation. A white point indicates where the aggregates were. * Indicates significant difference from all other groups (*p* < 0.05). Bars indicate mean ± SEM of three independent tests with three biological replicates each.

The measurement of the radius around the aggregates allowed the estimation of the migration and proliferation of cells on each surface, as shown in Figure 7. The radius of cells increased 2.6 times in groups C and PSHA and 3 times in the group MA and BE from day 2 to day 5. With two days, there was no significant difference (*p* > 0.05) between groups. However, on day five, the BE group had a significantly larger radius compared to all tested groups. It was possible to observe the fields used to measure the radius on Figure 7C, that showed where the aggregates were adhered (white point) and the number of cells around it with nuclei marked in blue with DAPI.

#### 3.3.4. Measurement of Cell Area

**Figure 8:**
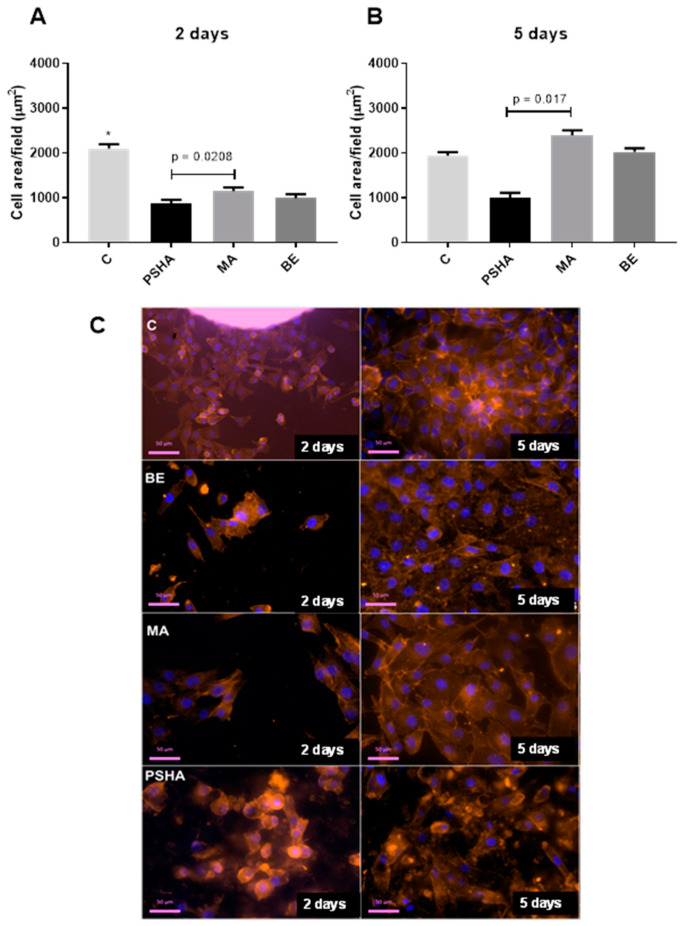
Cellular area measured in µm using Image-Pro Plus software for 2 days (**A**) and 5 days of exposure (**B**). On the second day, the MA group showed a difference compared to the PSHA group with *p* = 0.0208 and the C group showed a significant difference in relation to all groups (*). (**C**) vs. PSHA *p* < 0.0001, C vs. MA *p* = 0.0051, C vs. BE *p* < 0.0001. On the fifth day, the difference was between the MA and PSHA groups with *p* = 0.017. Panel C shows examples of the fields collected for each group for the analysis at 2 and 5 days. Bars indicate mean ± SEM of three independent tests with three biological replicates each.

The quality of adhesion was also assessed by measuring the cell spread over the different surfaces (Figure 8). The control group (Thermanox slides), as expected, had a greater cell spreading than all other groups (*p* < 0.05). On the second day of exposure, the PSHA group had cells with a smaller area than the MA group (*p* = 0.0208). On day five, the MA group showed a greater mean spreading area, but this difference was, again, only significant when compared to the PSHA group (*p* = 0.017). Panel C in Figure 8 shows representative fields collected for each group for the measurement of cell spread on days 2 and 5.

## 4. Discussion

Biomaterials for bone therapy in dentistry and implantology should present adequate biocompatibility and biofunctionality, compatible with the achievement of desirable clinical outcomes. Osteoconductivity is a very important feature for cells to migrate from bone tissue to the surface of the biomaterial, allowing osseointegration and avoiding implant movement. The in vitro evaluation of these properties requires constant research and development of new strategies, increasingly closer to clinical and physiological models for testing new health products. In this context, three-dimensional, organoid, and tissue-equivalent models were considered extremely promising to increase the prediction of in vitro tests. The main objective of the present work was to characterize and evaluate the use of a 3D model of osteospheres for the evaluation of different surfaces of titanium implants. For this, well-known titanium surfaces were used to analyze the model’s performance. The osteospheres demonstrated the ability to respond in different ways regarding migration, morphology, and biological behavior. The surface with sandblasting and acid etching performed better than surfaces with the hydroxyapatite coating by plasma spray and the machined (smooth) surface.

Titanium implants are widely used in dentistry for many rehabilitations because they are highly biocompatible, stable, and resistant to mechanical strength and corrosion. Osseointegration is a fundamental biological process for the success of an implant; therefore, several surface treatments have been developed in order to improve the adhesion of the implant to the host bone [25]. The physicochemical modifications performed by these treatments are capable of affecting the bone formation process. Topography directly affects protein adsorption processes and biological behavior such as adhesion, migration, proliferation and the differentiation of osteoblastic cells [26]. In this work, titanium discs with different surface treatments were evaluated: machined (MA), sandblasting and acid etching (BE), and plasma sprayed hydroxyapatite coating (PSHA). The physicochemical characterization showed that this study started with three surfaces with sufficiently distinct physicochemical properties. The FTIR and DRX showed the presence of calcium phosphates in the PSHA sample, while FTIR showed that the other two surfaces were constituted mostly by titanium. SEM and AFM confirmed the expected different topographies among the surfaces, with the MA being the least rough and the PSHA the roughest.

To test the applicability of the 3D model in cell/surface interaction studies, a model was produced using the liquid overlay methodology. These spheres presented a diameter in a range very common for three-dimensional models found in the literature [27,28,29]. The tendency towards diameters between 300 µm and 400 µm can be explained by the fact that, in the human body, the distance of a cell from the nearest capillary is rarely greater than 200 µm [30]. In this way, the cells are organized in the spheroid so that there are nutrients and oxygen even in the innermost layers. An arrangement allowing cell viability may explain the maintenance of the number of viable cells per spheroid around 1.9 × 10^4^ on the fourth day, a similar number of the initial seeding. The observation by scanning electron microscopy showed a uniformity of the aggregate in terms of surface regularity and dimensions, but heterogeneity in relation to the morphology of the superficial cells, with more spread cells and more rounded ones, which was indicative of cells at different stages of proliferation. Furthermore, it was possible to observe different intercellular interactions, a dense formation of microvilli, and spaces between cells, which allow the passage of oxygen and nutrients to the innermost layers. Such spaces were evidenced in the histological evaluation of the aggregates, which also confirmed the absence of an evident necrotic center, indicating that oxygen and nutrients managed to reach the innermost layers of the model. Through histological evaluations, it was also possible to observe the noticeable presence of calcium deposits on the fourth day of aggregate formation, evidenced by an alizarin red marking, evidence that this model was able to mature into a mineralized structure characteristic of bone tissue.

In vitro studies of cell/surface interactions often assess the density of adhered cells after uniform seeding over the biomaterial. In this study, a quantification by LDH allowed measuring the cell density of cells that covered the material, but also of those present in the spheres, while cell counting by fluorescence microscopy provided the detection only of cells that left the sphere and migrated or proliferated over the surface of the material. Comparing the results of these two assessments for the different titanium surfaces, we could observe that, while the total amount of cells was equivalent regardless of surface treatment, the number of adhered cells outside the aggregates was significantly different depending on the surface (*p* < 0.05). A possible interpretation is that these events are more related to cell migration than proliferation, as the number of cells that were adhered to the material increased distinctly between the surfaces, but the groups had similar numbers of cells every day, which indicates that there was an equivalent proliferation rate in all groups. This behavior allowed establishing an observable pattern by the increase in the halo of cells around the aggregates over time, where materials that presented a greater distance from the seeding point (halo radius) would indicate a greater migration/proliferation on the surface, suggesting osteoconductive properties. When an implant favors the adhesion and migration of osteoblastic cells, it enhances the covering with surrounding tissue and integrates it with the host bone.

The measurement of cell spreading on the test surface is a parameter used in several in vitro studies [31,32,33], as it may be indicative of stronger adhesion with possible impact on the osteoconductive properties of the biomaterial. In the present study, the surfaces with a greater spread (control group and machined titanium surface), were not necessarily those with more evidence of migration and proliferation. This indicates that the cellular responses leading to osteoconductivity are rather complex, and it is possible that an extremely strong interaction of a cell on a surface may contribute to the immobilization of the cell and, therefore, not resulting in a more efficient colonization of the entire surface. It is important to note that previous studies with 2D seeding could not easily detect this difference, as a well-occupied surface may be the result of well-dispersed seeding, and not the result of the colonization of cells from one point to another on the surface. Thus, the analysis of the properties of a biomaterial may consider the individual interactions of a cell, such as calculating the adhesion force and focal adhesion points [34], but it must also consider more dynamic properties such as those proposed in this work.

It is important to note that the data from the present study obtained by the three-dimensional model of osteospheres for the already well-studied implant surfaces corroborated the data from clinical studies found in the literature. The in vivo and in vitro results observed in the literature in general, demonstrate that titanium implants with sandblasting and acid etching (BE) treatment help to improve bone formation and mineralization [25,35,36,37], even though Ting et al. [38] found no significant difference in the adhesion of osteoblasts in vitro between surfaces treated with BE compared to the as-machined surface (MA). Long-term clinical results (over 10 years) showed that implants with BE treatment only had about a 3% chance of failure [39]. The results of the present work showed that the titanium discs with BE treatment had a greater migration/proliferation than the MA, PSHA, and even the control group (represented by a cell-adhesion-optimized surface). In fact, the use of machined surface implants in the clinic is limited. Due to its insufficient initial stability, this type of implant does not meet the needs of patients and is declining in the market [37]. In general, the results found in the literature demonstrate that MA implants have a lower performance than surfaces with some types of treatment [40,41,42,43]. The present results demonstrated that the MA surface had less cell migration, proliferation, and differentiation than the control and BE surface. Despite this, the cell area was greater on this surface compared to treated titanium surfaces. This same event was seen by other authors with MC3T3 murine preosteoblasts, which suggests that a lower roughness allows the cells to have a more stretched and flattened morphology [44].

The coating of implants with hydroxyapatite (HA) has the potential to increase osseointegration, as this is a biocompatible and bioactive substance that stimulates the production of several important molecules for the formation of new bone [45]. However, depending on the technique used to manufacture the implant, materials with HA coating do not always have great results when they reach the clinic. In this sense, it was reported that the Plasma Spray (PS) coating technique does not always provide a uniform coating with adequate crystallinity [46]. Controversial results are observed in the literature regarding the efficiency of titanium implants with HA coating using PS both in vivo and in vitro. Some authors demonstrate improved bone–implant binding and acceleration in the mineralization process at early implantation times [47,48,49]. However, there are in vivo [50] and in vitro [51] results suggesting a lower performance for these implants. Studies that refer to the use of PSHA implants in clinical practice report the failure of these implants in patients for reasons that include low mechanical strength at the interface between the HA coating and titanium. For these reasons, other techniques have been developed to overcome the limitations of PS [46,52]. At this juncture, we can observe that the present in vitro results, employing the 3D test model, were similar to those obtained in the clinic. The MG-63 bone cell spheroids employed in this study were produced through a simple method (agar overlay), which may produce a large amount of viable osteospheres, with a great uniformity and reproducibility, through a relatively easy protocol and at low costs. Similar 3D models have contributed to understand bone cell interactions with scaffolds for tissue engineering, such as 3D-printed polymer materials [53].

Among the limitations of the present study, we can include difficulties in processing for microscopy on low adherent surfaces, which impaired the observation of osteospheres over the MA surfaces and the restriction of the study to one type of graft material (titanium implants) which limited the overall indirectness of the conclusions. The use of transformed cells, with possible metabolic alterations when compared to primary cells, might also limit future studies that involve metabolic and complex molecular assessments. Nevertheless, it is worth noting that many similarities have already been reported in the literature between this lineage and normal osteoblasts, in addition to the fact that the use of transformed cells brings a greater reproducibility of interstudy results [54,55]. Finally, the limitation of in vitro studies in the extrapolation of results to the clinic must always be recognized, which demands the continuous development of new models. In this sense, the present study proposed to contribute a step forward in this search for the increased predictivity of in vitro models.

Therefore, before achieving further assumptions on the suitability of the proposed 3D model and endpoints employed in this study for the evaluation of biomaterials in general, tests need to be performed on other types of biomaterials, such as ceramics and bioactive glasses. Thereby, it will be possible to establish standardized parameters for materials used in bone therapy. In addition, other important parameters for the investigation of these new materials should be tested, such as the analysis of mineralization. This methodology can be widely used in dentistry if applied using other cell types that are of interest in the investigation of other types of materials. Therefore, improvements in this model in conjunction tests on more surfaces and the inclusion of new parameters can gradually contribute to the evolution of in vitro studies, gradually reducing the use of animals, contributing to a more predictive science, including following the new paradigms of research in the 21st century.

## 5. Conclusions

A three-dimensional model of human bone cell spheroids produced by agar overlay was able to identify different interactions regarding spreading, attachment, and migration over different titanium implant surfaces. The titanium sandblasted and acid etching surface performed better than the surfaces with hydroxyapatite coating by Plasma Spray and the machined surface, as expected for the clinical reports found in the literature. These results suggested the model to be a promising tool for the qualitative and quantitative in vitro analysis cell/surface interactions with biomaterial surfaces related to osteoconductivity which are of relevance on the development of materials for bone therapy and bioengineering.

## Figures and Tables

**Table 1:** Comparative analysis between the PSHA, MA and BE surfaces regarding mean roughness (Sa), mean square roughness (Sq), maximum pit depth (Sv), and maximum height (Sz).

Surface	Mean Roughness (Sa)	Mean Square Roughness (Sq)	Maximum Pit Depth (Sv)	Maximum Height (Sz)
PSHA	0.344 ± 0.063	0.431 ± 0.059	1.49 ± 0.090	2.516 ± 0.67
MA	0.055 ± 0.043	0.067 ± 0.048	0.175 ± 0.080	0.338 ± 0.151
BE	0.251 ± 0.181	0.324 ± 0.234	0.875 ± 0.641	2.110 ± 1.462

**Table 2:** Values of diameter and aspect of osteospheres seeded at different cell densities, on the 4th day of formation.

Cell Density (At Seeding)	20,000	30,000	40,000
Diameter (μm)	329.5 ± 11.2	429.4 ± 35.01	507.2 ± 43.6
Aspect (H/W)	1.2 ± 0.1	1.2 ± 0.06	1.2 ± 0.08

(H/W) = height/width; (n) = 20 aggregates.

## Data Availability

The authors will provide any raw data upon request.
